# Revisiting Middle East Respiratory Coronavirus (MERS-CoV) Outbreak Chest Radiographic Initial Findings, Temporal Progression, and Correlation to Outcomes: A Multicenter Study

**DOI:** 10.7759/cureus.24860

**Published:** 2022-05-09

**Authors:** Amr M Ajlan, Nesreen H Abourokbah, Samirah Alturkistani, Rayan A Ahyad, Ahmed Alharthy, Majed Ashour, Ghassan Wali, Tariq A Madani

**Affiliations:** 1 Department of Radiology, King Abdulaziz University Faculty of Medicine, Jeddah, SAU; 2 Medical Imaging, King Abdulaziz Medical City, Jeddah, SAU; 3 Department of Radiology, King Faisal Specialist Hospital and Research Centre, Jeddah, SAU; 4 Internal Medicine, King Faisal Specialist Hospital and Research Centre, Jeddah, SAU; 5 Department of Medicine, King Abdulaziz University Faculty of Medicine, Jeddah, SAU

**Keywords:** ground-glass opacities, consolidation, chest x-ray, mers-cov, middle east respiratory coronavirus

## Abstract

Objectives

Accounts of initial and follow-up chest X-rays (CXRs) of the Middle East respiratory coronavirus (MERS-CoV) patients, and correlation with outcomes, are sparse. We retrospectively evaluated MERS-CoV CXRs initial findings, temporal progression, and outcomes correlation.

Materials and methods

Fifty-three real-time reverse-transcriptase-polymerase chain reaction (rRT-PCR)-confirmed MERS-CoV patients with CXRs were retrospectively identified from November 2013 to October 2014. Initial and follow-up CXR imaging findings and distribution were evaluated over 75 days. Findings were correlated with outcomes.

Results

Twenty-two of 53 (42%) initial CXRs were normal. In 31 (68%) abnormal initial CXRs, 15 (48%) showed bilateral non-diffuse involvement, 16 (52%) had ground-glass opacities (GGO), and 13 (42%) had peripheral distribution. On follow-up CXRs, mixed airspace opacities prevailed, seen in 16 (73%) of 22 patients 21-30 days after the initial CXRs. Bilateral non-diffuse involvement was the commonest finding throughout follow-up, affecting 16 (59%) of 27 patients 11-20 days after the initial CXRs. Bilateral diffuse involvement was seen in five (63%) of eight patients 31-40 days after the initial CXRs. A bilateral diffuse CXR pattern had an odds ratio for mortality of 13 (95% CI=2-78) on worst and 18 (95% CI=3-119) on final CXRs (P-value <0.05).

Conclusion

Initially, normal CXRs are common in MERS-CoV patients. Peripherally located ground-glass and mixed opacities are common on initial and follow-up imaging. The risk of mortality is higher when bilateral diffuse radiographic abnormalities are detected.

## Introduction

The Middle East respiratory coronavirus (MERS-CoV) emerged in Saudi Arabia in 2012 and since has spread to 27 countries [1,2]. Up to the end of 2019, the confirmed number of MERS-CoV cases was 2499, with 858 deaths [1]. MERS-CoV, with camels acting as intermediate hosts and a primary source of infection, is mainly spread in the community and healthcare facilities via human-to-human transmission, causing major outbreaks [1,3,4]. Although the last outbreak occurred in Korea in 2015 [3], the virus still causes predominantly seasonal clusters in Saudi Arabia, the Middle East, and other countries [5].

Both coronaviruses currently coexist in the Middle East and around the globe in symptomatic and asymptomatic individuals [6-9]. The case-fatality rate of MERS-CoV is higher compared to coranovirus disease 19 (COVID-19) [1,9], despite remarkably similar imaging manifestations of both viral infections. [7-9]. The utility of chest X-rays (CXRs) in investigating MERS-CoV is practically convenient, yet the number of dedicated studies addressing the CXR imaging findings is limited, especially when compared to that of their CT features [10-12].

We retrospectively revisited the local 2013-2014 MERS-CoV outbreak; aiming to identify the radiographic appearance on the initial CXRs and temporal progression over follow-up studies. We also studied imaging predictors of final patients’ outcomes. This article was previously posted to the research square preprint server on June 18, 2020.

## Materials and methods

Subjects

The local research ethics committees of three participating hospitals approved this retrospective study, and the consent form was waived (reference numbers: 108-14, IRBC/309/14, and RC-J/217/35). The electronic archives were searched from November 2013 to October 2014 for all cases with a confirmed diagnosis of MERS. Case confirmation was based on at least one positive real-time reverse-transcriptase-polymerase chain reaction (rRT-PCR) respiratory sample, by targeting the MERS-CoV RNA upstream region of the E gene and the open reading frame ORF1a and ORF1b regions. Five patients with no chest radiographic imaging and three patients younger than 18 years of age were excluded. Seven patients included in this study were previously reported, but for CT imaging features and not for CXR findings (interpreters in our current study were blinded to CT in all patients) [13]. The clinical, laboratory and imaging findings and outcomes were analysed. Diabetes mellitus, hypertension, chronic lung diseases, cardiac diseases, chronic renal failure, neurological deficits, pregnancy, or immunocompromised status were recorded as comorbidities. Laboratory-confirmed superadded infection from other organisms was recorded. For outcome data analysis, patients were divided into deceased versus survived; the latter include those transferred to other institutions with no further follow-up data.

Image analysis

Standard-technique chest radiographs were obtained in upright posteroanterior or portable anteroposterior projections using the following systems: Carestream DirectView system (Carestream, Rochester, NY USA), OmniDiagnost (Philips, Amsterdam, Netherlands), Ysio (Siemens, Munich, Germany), Mobvision DR unit (Almana Medical Imaging, Al-Khubar, Eastern Region, Saudi Arabia), GE Definium AMX 700 (GE, Boston, MA, USA), and Carestream DRX-Revolution (Carestream, Rochester, NY USA). Three fellowship-trained thoracic radiologists, each with 11-12 years of practical experience, reviewed the frontal chest radiographs on Sectra IDS7 (Sectra, Linköping, Sweden) and Agfa IMPAX (Agfa Healthcare, Mortsel, Belgium) picture archiving and communication system workstations.

Applied terminology was in line with the Fleischner Society terms [14]. Airspace opacities were classified as ground-glass opacities (GGO) if underlying lung markings were not obscured, as consolidation if underlying lung markings were obscured, and as mixed opacities, if both patterns coexisted. Interstitial opacities were divided into reticular if linear, nodular if rounded and reticulonodular if both patterns coexisted. The presence and laterality of pneumothoraces and pleural effusions, as well as the presence of cavities (i.e. rounded or oval air- or fluid level-containing lucencies), were sought.

Lung involvement by airspace opacities was categorised as being unilateral or bilateral. The abnormality was assigned craniocaudal or transverse distributions, whenever possible, whether unilateral or bilateral, focal or diffuse. The predominant craniocaudal distribution was categorised as being in the upper lung (i.e. above the hilum), in the lower lung (i.e. below the hilum) or nonspecific (i.e. with equal upper and lower lung involvement). The predominant transverse distribution was categorised as being central (i.e. perihilar), peripheral (i.e. subpleural), both central and peripheral (i.e. if perihilar and subpleural locations are distinctly seen on the same radiograph) or nonspecific (i.e. scattered or diffuse opacities with no distinctive central or peripheral predominance). The abnormality was considered ‘focal’ if it was unilateral and predominantly confined to the upper or lower lung; and further assigned the following locations: right upper, right lower, left upper or left lower lung.

All available initial and follow-up CXRs were evaluated for the features mentioned above. The presentation CXR (i.e. within the first day) was designated as the ‘initial’ examination (period I) and was assessed separately. The follow-up radiographs were assessed according to the following periods: >1-5 follow-up days (period II), 6-10 follow-up days (period III), 11-20 follow-up days (period IV), 21-30 follow-up days (period V), 31-40, 41-50 follow-up days, (period VI) and 51-75 follow-up days (period VII). For each patient in each period, the most predominant and persistent findings and distribution were subjectively recorded collectively for all follow-up CXRs performed during that same period. The final follow-up chest radiograph was categorised as normal in a discharged patient, improved in a discharged patient, abnormal in an expired patient or abnormal in a patient transferred to a non-participating isolation institution.

An overall imaging pattern was summarised for each patient in each period, from better to worse, as follows: normal (i.e. no abnormalities detected), focal opacity (i.e. unilateral airspace involvement of an upper or lower lung location), bilateral non-diffuse opacities (i.e. bilateral airspace involvement of clear upper or lower and central or peripheral predominance) or bilateral diffuse opacities (i.e. homogenous or heterogeneous bilateral airspace involvement with no clear craniocaudal or transverse predominance). Additionally, each patient was assigned the worst imaging pattern of involvement according to the period for which it was first seen. For example, bilateral non-diffuse opacities were considered worse than a focal opacity (If the patient developed both patterns during the study duration, the period with the worst imaging pattern was considered that of when the bilateral non-diffuse opacities were first seen).

Statistical analysis

Statistical analyses were performed with the Statistical Package for Social Sciences version 21.0 for Windows (SPSS Inc., Chicago, IL, USA). Data are presented as frequencies and percentages for categorical variables, and as means ± standard deviations (SDs) for continuous variables. Independent t-test was used to compare means and SDs of age (in years) of survived versus deceased patients. Factors of mortality were analysed by comparing means ± SDs of age between survived and deceased patients using independent t-test, and by comparing the percentage of survived and deceased patients for other factors using χ2 or Fisher’s exact tests, as appropriate. Multivariate binary regression was performed to determine whether a bilateral diffuse pattern on worst and final CXRs-beside other covariates-was an independent factor of mortality; with results presented as odds ratios (OR) with 95% CI. For all calculations, a P-value of <0.05 was considered statistically significant.

## Results

Patients’ results

Patients’ demographics, clinical characteristics and hospital course are detailed in Table 1, and laboratory results are detailed in Table 2. Fifty-three hospitalised patients constituted the study cohort, 33 (62%) of whom were males (Table 1). The age range of the total population was 23-76 years (mean and SD, 43.7 ± 15.4 years). Twenty-one (40%) patients had one or more comorbidity. Cough and fever were the most frequent symptoms, each encountered in 39 (74%) patients. The initial symptoms were non-respiratory complaints in nine (17%) patients. Superadded bacterial infection occurred in 20 (38%) patients. Twenty-six (49%) patients required respiratory support during any of the studied periods. Twenty-nine (55%) patients were discharged, 15 (28%) patients died during admission, and nine (17%) patients were transferred to non-participating institutions.

**Table 1:** Patients’ demographics, clinical characteristics and hospital course ^a^Calculations are means (and standard deviations), whereas the rest of the table parameters are calculated as frequencies (and percentages). ^b^Frequencies (and percentages) are higher than the population sum total (and 100%) due to parameter overlap in patients^. c^Patients eventually transferred to healthcare centre other than the three hospitals subject to the study. ICU: intensive care unit; BiPAP: bi-level positive airway pressure; ECMO: extracorporeal membrane oxygenation

Parameter	Value
Demographics and clinical characteristics	
Age (years)^ a^	43.7 (±15.4)
Age category (years)	
≤44	31 (58%)
>44	22 (42%)
Sex	
Male	33 (62%)
Female	20 (38%)
Contact history	
None	21 (40%)
Confirmed	23(43%)
Unclear in records	9 (17%)
Significant comorbidities	
Present	21 (40%)
None	32 (60%)
Presenting symptoms^b^	
Cough	39 (74%)
Fever	39 (74%)
Myalgia	19 (36%)
Abdominal pain	10 (19%)
Diarrhoea	7 (13%)
Dyspnoea	28 (53%)
Chest pain	8 (15%)
Rhinitis	4 (8%)
Nausea and vomiting	7 (13%)
Headache	12 (23%)
Haemoptysis	5 (9%)
Type of initial symptoms at presentation	
Respiratory	44 (83%)
Non-respiratory	9 (17%)
Hospital course	
Period from symptoms onset to hospital presentation (days)^a^	5.8 (± 4.6)
Duration of admission (days)^a^	22.4 (±27.9)
Superadded infection	
Yes	33 (62%)
No	20 (38%)
Admission location	
ICU	29 (55%)
Others	24 (45%)
Respiratory support	
None	27 (51%)
Intubation	7 (13%)
BiPAP	17 (32%)
ECMO	2 (4%)
Clinical outcome	
Discharged	29 (55%)
Transferred to another facility^c^	9 (17%)
Death	15 (28%)

**Table 2:** Patients’ laboratory results at presentation Note: Values are represented in frequency and percentage. ^a^Frequencies (and percentages) are less than the population total (and 100%) due to parameter unavailability/uncertainty in the hospital records.

Parameter		Value
Initial specimen type^a^	Nasopharyngeal swab	43 (83%)
	Tracheal aspirate	6 (12%)
	Bronchoalveolar lavage	2 (4%)
	Induced sputum	1 (2%)
White blood cells (Normal = 4-11 x 10^3^ cells per mm^3^ of blood)	Normal	23 (43%)
	Elevated	10 (19%)
	Low	20 (38%)
Lymphocytes^a^ (Normal = 1.5-4 x 10^3 ^cells per mm^3^ of blood)	Normal	10 (19%)
	Elevated	1 (2%)
	Low	41 (79%)
Eosinophils (Normal = 1-7 x 10^3^ cells per mm^3^ of blood)	Normal	9 (17%)
	Elevated	40 (76%)
	Low	4 (8%)
Haemoglobin (Normal = 13.0-18.0 gm/dL)	Normal	27 (51%)
	Low	26 (50%)
Platelets (Normal = 150-450 x 10^3^ cells per mm^3 ^of blood)	Normal	23 (43%)
	Elevated	3 (6%)
	Low	27 (51%)
Lactate dehydrogenase^a^ (Normal = 125-243 U/L)	Normal	6 (16%)
	Elevated	31 (81%)
	Low	1 (3%)
Alanine aminotransferase^a^ (Normal = 5-55 U/L)	Normal	27 (54%)
	Elevated	22 (44%)
	Low	1 (2%)
Aspartate aminotransferase^a^ (Normal = 5-34 U/L)	Normal	12 (24%)
	Elevated	37 (73%)
	Low	2 (4%)
Creatine kinase^a^ (Normal = 30-200 IU/L)	Normal	11 (31%)
	Elevated	21 (60%)
	Low	3 (9%)
Creatinine^a^ (Normal = 60-115 μmol/L)	Normal	29 (56%)
	Elevated	18 (35%)
	Low	5 (10%)

Imaging findings

General Findings

Over the total follow-up period, a sum of 692 CXRs was performed and analysed (range of 1-55 per patient; mean and SD, 7 ± 14 CXRs). Out of 46 patients with a known duration from symptoms onset to the time of obtaining the initial CXR, 17 (37%) had normal initial CXRs; obtained within a period of one to 34 days (mean and SD, 5.5 ± 7.7 days). According to imaged period, chest radiographic detailed findings are summarised in Table 3, while radiograph patterns, radiographic abnormalities distributions and clinical status by follow-up period are summarised in Table 4.

**Table 3:** Patients’ detailed chest radiograph findings according to imaging periods (a) Note: Values are represented in numbers (n) and percentages (%) unless otherwise indicated. ^a^Periods are relative to the time of obtaining the initial CXR. ^b^Period I is within the first day of obtaining the initial CXR. ^c^Periods with increasing instead of decreasing patients’ numbers due to fluctuation in the number of imaged patients per period. CXR: chest X-ray

	I (Initial CXR)^b^		II (Days 2-5)		III (Days 6-10)		IV (Days 11-20)		V (Days 21-30)		VI (Days 31-40)		VII (Days 41-50)		VIII (Days 51-75)		
Number and % of CXR																	
Number of imaged patients (n=53)	53	100%	36	68%	39	74%^c^	27	51%	22	42%	8	15%	2	4%	3	6%^c^	
Number of CXR (Mean ±SD)	12.7	±14.3	2.5	±2.2	3.1	±2.6	5.5	±4.3	5.6	±4	3.9	±2.9	3	±2.6	4.7	±4	
*Findings*																	
Airspace opacity																	
Consolidation	6	11%	5	14%	4	10%	4	15%	0	0%	0	0%	0	0%	0	0%	
Ground-glass opacities	16	30%	5	14%	9	23%	6	22%	2	9%	0	0%	1	50%	1	33%	
Both	9	17%	20	56%	21	54%	15	56%	16	73%	5	63%	1	50%	1	33%	
Focal opacity																	
Right upper	1	2%	0	0%	0	0%	0	0%	0	0%	0	0%	0	0%	0	0%	
Right lower	6	11%	4	11%	5	13%	2	7%	0	0%	0	0%	0	0%	0	0%	
Left upper	2	4%	1	3%	0	0%	0	0%	0	0%	0	0%	0	0%	0	0%	
Left lower	4	8%	2	6%	1	3%	2	7%	1	5%	0	0%	0	0%	0	0%	
Interstitial opacity																	
Reticular	1	2%	1	3%	5	13%	4	15%	6	27%	3	38%	0	0%	0	0%	
Nodular	0	0%	0	0%	0	0%	0	0%	0	0%	0	0%	0	0%	0	0%	
Reticulonodular	0	0%	0	0%	0	0%	2	7%	3	14%	2	25%	1	50%	1	33%	
Bronchial wall thickening	2	4%	1	3%	3	8%	6	22%	5	23%	2	25%	0	0%	1	33%	
Pleural effusion																	
Right	1	2%	3	8%	0	0%	1	4%	1	5%	0	0%	0	0%	0	0%	
Left	2	4%	0	0%	3	8%	1	4%	0	0%	0	0%	0	0%	0	0%	
Both	3	6%	3	8%	5	13%	4	15%	5	23%	4	50%	0	0%	0	0%	
Pneumothorax																	
Right	0	0%	0	0%	2	5%	1	4%	1	5%	0	0%	0	0%	0	0%	
Left	0	0%	0	0%	0	0%	1	4%	2	9%	0	0%	0	0%	0	0%	
Both	0	0%	0	0%	0	0%	1	4%	1	5%	0	0%	0	0%	0	0%	

**Table 4:** Summarised chest radiograph patterns, abnormalities distribution and clinical status by follow-up periods (a) Note: Values are represented in numbers (n) and percentages (%) unless otherwise indicated. a Periods are relative to the time of obtaining the initial CXR. b Period I is within the first day of obtaining the initial CXR. CXR = chest X-ray

	I (Initial CXR)^b^ N=53		II (Days 2-5) N=36		III (Days 6-10) N=39		IV (Days 11-20) N=27		V (Days 21-30) N=22		VI (Days 31-40) N=8		VII (Days 41-50) N=2		VIII (Days 51-75)		
	N=3		
Summarised CXR pattern																	
Normal	22	42%	6	17%	5	13%	1	4%	4	18%	2	25%	0	0%	1	33%	
Focal opacity	13	25%	7	19%	6	15%	4	15%	1	5%	0	0%	0	0%	0	0%	
Bilateral non-diffuse opacities	15	28%	17	47%	21	54%	16	59%	10	46%	1	13%	1	50%	1	33%	
Bilateral diffuse opacities	3	6%	6	17%	7	18%	6	22%	7	32%	5	63%	1	50%	1	33%	
Distribution																	
Axial distribution																	
Central	1	2%	0	0%	0	0%	0	0%	0	0%	0	0%	0	0%	0	0%	
Peripheral	13	25%	5	14%	7	18%	3	11%	2	9%	0	0%	1	50%	1	33%	
Both	7	13%	6	17%	8	21%	9	33%	6	27%	1	13%	0	0%	0	0%	
Unclassified	10	19%	19	53%	19	49%	14	52%	10	46%	5	63%	1	50%	1	33%	
Craniocaudal distribution																	
Upper	3	6%	1	3%	0	0%	0	0%	0	0%	0	0%	0	0%	0	0%	
Lower	23	43%	22	61%	24	62%	18	67%	10	46%	1	13%	1	50%	1	33%	
Unclassified	5	9%	7	20%	10	26%	8	30%	8	36%	5	63%	1	50%	1	33%	
Side of involvement																	
Unilateral	13	25%	7	19%	6	15%	4	15%	1	5%	0	0%	0	0%	0	0%	
Bilateral	18	34%	23	64%	28	72%	22	82%	17	77%	6	75%	2	100%	2	67%	
Clinical status																	
Hospitalised	47	89%	32	89%	27	69%	19	70%	8	36%	2	25%	2	100%	0	0%	
Discharged (with normal CXR)	4	8%	2	6%	3	8%	2	7%	3	14%	1	13%	0	0%	1	33%	
Transferred (with abnormal CXR)	1	2%	1	3%	2	5%	1	4%	2	9%	0	0%	0	0%	2	67%	
Died	0	0%	1	3%	2	5%	3	11%	5	23%	4	50%	0	0%	0	0%	
Discharged (improved CXR)	1	2%	0	0%	5	13%	2	7%	4	18%	1	13%	0	0%	0	0%	

Initial CXR Imaging Findings

**Figure 1:**
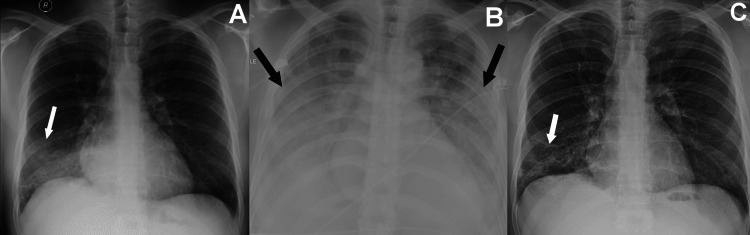
CXRs of a 27-year-old medical professional male survivor of MERS-CoV infection (A) Initial CXR obtained at 10 days from symptoms onset, with focal right lower lung ground-glass opacity (arrow); (B) Worst CXR first seen five days from time of obtaining the initial CXR (i.e. study period II), showing bilateral non-diffuse pattern of lower-lung-predominate mixed ground-glass and consolidative opacities (arrows); (C) Final CXR obtained after 73 days from the time of obtaining the initial CXR (i.e. study period VI), showing right lower lung ground-glass opacities and reticulations (arrow). MERS-CoV: Middle East respiratory coronavirus

**Figure 2:**
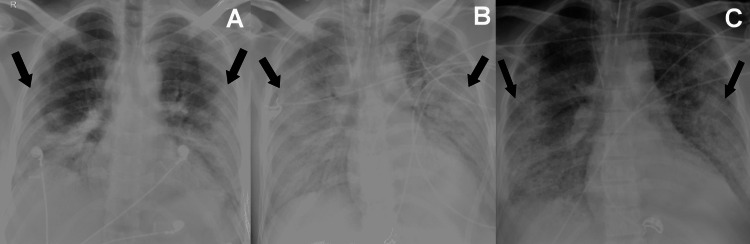
CXRs of a 44-year-old male succumbing to MERS-CoV infection (A) Initial CXR obtained after eight days from symptoms onset, with bilateral non-diffuse pattern of peripheral and lower-lung-predominate mixed ground-glass and consolidative opacities (arrows); (B) Worst CXR, first seen five after the initial CXR (i.e. study period II), showing bilateral diffuse pattern of mixed ground-glass and consolidative opacities (arrows); (C) Final CXR, obtained 15 days after the initial CXR (i.e. study period IV), showing bilateral non-diffuse pattern of peripheral and lower-lung-predominate mixed ground-glass and consolidative opacities (arrows). MERS-CoV: Middle East respiratory coronavirus

**Figure 3:**
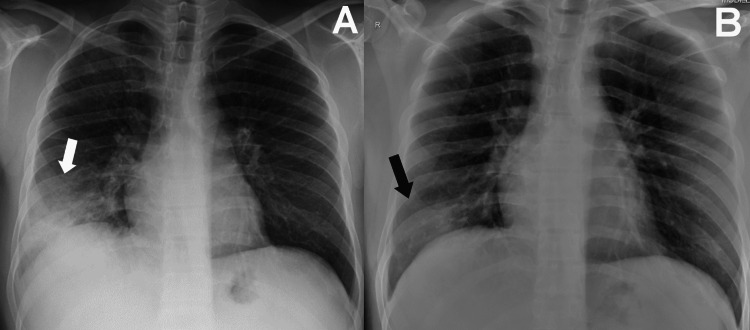
CXRs of a 31-year-old male survivor of MERS-CoV infection (A) Initial CXR, obtained after eight days from symptoms onset, showed the worst observed pattern in this patient, with focal right lower lung ground-glass opacity (arrow); (B) Final CXR, obtained after eight days from the initial CXR (i.e. study period III), showing residual focal right lower lung ground-glass opacity (arrow). MERS-CoV: Middle East respiratory coronavirus

**Figure 4:**
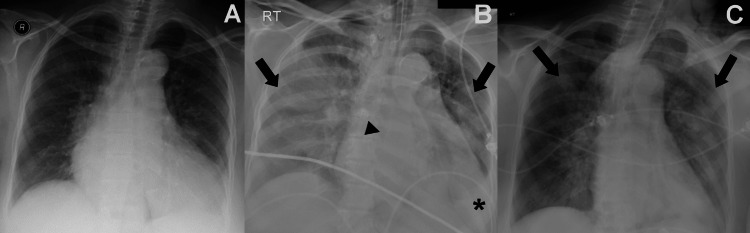
CXRs of a 56-year-old female known for cardiac comorbidity, succumbing to MERS-CoV infection (A) Initial CXR obtained at two days from symptoms onset, with normal lung and baseline cardiomegaly; (B) Worst CXR, first seen three days after the initial CXR (i.e. study period II), showing bilateral diffuse pattern of heterogenous ground-glass opacities (thick arrows), focal left lower lobe consolidation (thin arrow) and left pleural effusion (arrowhead); (C) Final CXR, obtained eight days after the initial CXR (i.e. study period III), showing bilateral diffuse pattern of residual heterogenous ground-glass opacities (arrows). MERS-CoV: Middle East respiratory coronavirus

On 53 initial CXRs, 31 (58%) patients had abnormal CXRs (Figure 1). In the 31 abnormal initial CXRs, findings were bilateral in 18 (58%) patients (Figure 2), lower lung distribution in 23 (74%) patients, peripheral distribution in 13 (42%) cases and combined peripheral and central distribution in seven (23%) cases. GGOs were the most common finding, observed in 16 (52%) of 31 patients, followed by mixed airspace opacities in nine of 31 (29%) cases. Focal opacities were found in 13 (42%) of 31 patients (Figure 3), preferentially involving the lower lobes (10 cases; 77%). Interstitial (i.e. reticular) opacities were only found in one (2%) of 31 patients (Figure 4). Pleural effusion was found in six (19%) of 31 cases. Initial CXR findings of the nine patients who presented with non-respiratory symptoms showed a normal image in three (33%), focal opacities in three (33%), bilateral non-diffuse opacities in two (22%) and bilateral diffuse opacities in one (11%) (results not presented in tables). No cavities were detected.

Evolution of CXR Imaging Findings

The subsequent imaging periods were marked by increasing frequency of bilateral involvement, reaching 30 of 39 (77%) patients imaged at period III, seven (18%) of whom had a bilateral diffuse pattern. The proportionally largest percentage of bilateral diffuse involvements was encountered during period V (seven of 22 patients; 32%) and period VI (five of eight patients; 63%). A bilateral non-diffuse pattern predominated otherwise in most periods, especially in periods II and V, where it constituted 46 and 59% of the imaged patients in each period; respectively. As for the type of follow-up findings, mixed airspace opacities predominated throughout, reaching up to 16 (73%) of 22 patients imaged at period V. Interstitial opacities became relatively more frequent at periods III and VI, with the highest frequency being that of nine (41%) of 22 patients encountered at period V. Five (9%) of 53 patients developed a pneumothorax at different periods of the studied duration. Pleural effusions were infrequent. No cavities were detected.

Worst CXR Imaging Findings

Of the 53 patients, the worst CXR imaging finding was first encountered on the initial CXR in 26 (49%), period III in nine (17%), period IV in 11 (21%), period V in three (6%), period VI in three (6%) and period VII in one (2%) patients.

Predictors of patients’ outcome

Mortality was significantly associated with both worst and final CXR patterns. That is, no mortality was observed in the case of normal or focal opacity CXR patterns images in both the worst and last CXRs. However, a bilateral diffuse CXR pattern in the worst and last CXRs was associated with 62.5% and 76.9% mortality, respectively (P-value of 0.001). Additionally, patients who died were significantly older (>44 years) compared to survivors at follow-up (55 ±16 versus 39 ±13 patients, respectively), with a P-value of 0.001. The mortality rate was also significantly higher in cases with superadded infection compared to cases without superadded infection (50.0% versus 15%, respectively), with a P-value of 0.011. No significance was elucidated for either gender or comorbidity.

On separate multivariate regression models, a bilateral diffuse CXR pattern in both worst (OR=13, P-value=0.006) and last (OR=18, P-value=0.002) CXRs were independently associated with patient death; alongside an age >44 years old, which was highly predictive of death in both groups. However, superadded infection showed no statistical significance on multivariant regression analysis.

## Discussion

Only a few studies have addressed CXR findings of MERS-CoV [10,12,15,16]. This current study and that of Das et al. [10] are, to the best of our knowledge, the only publications describing the temporal radiographic findings in a relatively large hospitalised population sample. We addressed CXR imaging findings over various periods. Compared to Das et al. [10], we evaluated patients over a more extended follow-up period and a larger number of analysed CXRs. We also categorised the overall CXR imaging into simple patterns, in a way that could be easier for physicians to digest and potentially correlate to outcomes.

We encountered 42% of patients with initially normal CXRs, a percentage comparable to that of the study by Hamimi [12], but different from the 17% who reported otherwise [2,8,10]. Our cohort showed almost equal numbers of focal and bilateral non-diffuse initial CXR patterns. In line with other publications [2,8,10], the normality of CXRs decreased as patients were followed, with an increasing number of bilateral non-diffuse or diffuse lung involvement. We noticed that the worst CXR progression occurred between a week and a month from the time of obtaining the initial CXR.

GGO, whether pure or mixed with consolidation, was the most common CXR finding in our study. As the abnormalities progressed over time, superimposed consolidation appeared or progressed. Whether abnormalities were seen on initial or follow-up CXRs, the most common distribution was that of bilateral peripheral location, with or without perihilar involvement. The predilection for such peculiar distribution has previously raised the suggestion that the lung acutely responds to unknown novel pathogens in the form of an acute organising pneumonia reaction [13,17,18].

Pleural effusions, pneumothoraces, cavities and bronchial wall thinking were all rarely seen in our study, and are not common in the literature on CXR imaging of MERS-CoV [2,8,10,13]. A noteworthy observation is that interstitial opacities were also uncommon in our study and other reports. However, reticulations were observed in some patients where longer-term follow-up imaging was obtained [8,13,19]. There is a possibility that long-term sequelae of coronaviruses pneumonia include residual fibrosis [8,13].

Prior MERS-CoV research has shown that the risk of mortality significantly increases in proportion to the extent of lung involvement [2,8,10,19]. Such publications are in concordance with our observation that a bilateral diffuse pattern of lung abnormalities, as observed on the worst or finally imaged CXR, was independently related to higher death rates. On the other hand, no death occurred in patients whose worst or finally imaged CXRs were normal or focally abnormal. Further, the risk of MERS-CoV mortality in our group was found to be highest when patients were >44 years old.

The expected variability of sampled CXRs per various periods and the decreasing number of CXRs obtained as time progressed reduced the studied samples per specific periods. Furthermore, the study size is considered relatively small for deriving generalised outcome data conclusions. This point is even more relevant when considering the potentially confounding factor of superadded infection, which may by itself, cloud CXR interpretations. Another limitation is that a proportion of patients who were eventually transferred to other institutes were not included in the study scope but were considered survivors.

## Conclusions

A substantial number of MERS-CoV cases may have a normal initial CXR. When abnormal, bilateral lower-lung-predominant ground-glass or mixed opacities on CXRs were the most prevalent imaging appearance. Worsening CXRs progression occurred within a week to a month from the initial imaging. Bilateral diffuse lung involvement is an independent risk for higher mortality, worsened by older patients.
